# Fabrication of Magnetic Catalyst Fe_3_O_4_-SiO_2_-V_3_ and Its Application on Lignin Extraction from Corncob in Deep Eutectic Solvent

**DOI:** 10.3390/polym13101545

**Published:** 2021-05-12

**Authors:** Maonan Yuan, Zhen Wang, Yu Liu, Guihua Yang

**Affiliations:** State Key Laboratory of Biobased Material and Green Papermaking, School of Environmental Science and Engineering, Qilu University of Technology (Shandong Academy of Sciences), Jinan 250353, China; maonanyuan@126.com (M.Y.); liuy@qlu.edu.cn (Y.L.); ygh@qlu.edu.cn (G.Y.)

**Keywords:** lignin extraction, magnetic catalyst, deep eutectic solvent, corncob

## Abstract

Fe_3_O_4_-SiO_2_-V_3_ was prepared by deposited H_6_PMo_9_V_3_O_40_ on Fe_3_O_4_-SiO_2_ and employed as a catalyst to extract lignin from corncob in deep eutectic solvent (choline chloride/lactic acid = 1/10). Batch experiments were conducted in an autoclave under the conditions of 500 kPa, 90–130 °C and 15 h, while the dosage of the catalyst was set as a variable. Results indicated that the catalyst could effectively improve the qualities of the lignin, while the characteristics of the lignin showed prominent changes with the participation of the catalyst: the extraction rate increased from 71.65% to 98.13%, the purity was improved from 85.62% to 97.09%, and both the number average molecular weight and the weight average molecular weight also decreased significantly. Besides, the molecular distribution of the lignin achieved from the CC-LA-Fe-Si-V_3_ reaction system was found to be more highly concentrated (Polydispersity index = 1.746). Results from 2D NMR HSQC analysis indicated that lignin fractions achieved from the CC-LA-Fe-Si-V_3_ system showed distinct destruction involving C_2_-H_2_ in guaiacyl units (G), C_5_-H_5_ in guaiacyl units (G), and the C_γ_-H_γ_ in γ-hydroxylated β-O-4′ substructures, but little changes in the C_γ_-H_γ_ in phenylcoumaran substructures.

## 1. Introduction

As the second most abundant biopolymer in nature, lignin is the only non-petroleum resource that provides renewable aromatic compounds [1]. Annually, more than 50 million tons of lignin-based materials are produced, mainly by the pulp and paper industries [2]. However, less than 2% of them can be valorized and the vast majority of them are burned and used for energy recovery at the pulp mills [3]. Since the valorization of lignin highly affects the performance of biorefinery processes, it is imperative to develop various processes for lignin valorization. However, the extraction methods seriously restrain the subsequent bio-refinery processes. Lignins derived from traditional kraft pulping, sulfite cooking, and organosolv pulping usually exhibit unsatisfactory performance due to their low extraction rate, low purity, unevenly molecular weight distribution, poor reaction activity, and condensed structures [4]. The characteristics and extraction rate of lignin have significant influence on lignin valorization [5].

Deep eutectic solvents (DESs) are a new class of ionic liquids based on eutectic mixtures of hydrogen bond acceptor (HBA) and hydrogen bond donor (HBD). Compared with traditional ionic liquids, DESs have the advantages of low costs, biopolymer dissolution capability, biodegradability, nontoxicity, polarity, and recyclability, which make them appropriate media for biomaterials processing. In previous research, DESs exhibited perfect performance in the field of lignocellulosic biomass treatment [6]. They are reported to be beneficial to the processes of delignification, components dissolubility, conversion of lignocellulosic waste, and cellulose treatment (dissolution, modification, etc.) [7]. Among them, CC/lactic acid, betaine/lactic acid, and some amino acid-based DESs (lactic acid/proline, malic acid/proline, malic acid/glycine) were found to have advantages in dissolving lignin and to exhibit tiny cellulose solubility. The lignin extraction rate of wheat straw is about 58% in the CC/oxalic acid dihydrate (1:1) system [6]. The lignin extraction efficiency of the ChCl/oxalic acid dihydrate system can reach 80% under the conditions of 110 °C and 9 h with the assistance of microwaves (800 W, 80 °C). The obtained lignin fraction (LF) possesses a low molecular weight (913 g·mol^−1^), low polydispersity (1.25), and high purity [8]. Abdulmalek and his co-workers reported that around 47% lignin and 87% hemicellulose were removed from oil palm trunk fibers in the ethylammonium chloride/ethylene glycol (molar ratio 1:2) system, and that the obtained pretreated fibers showed excellent enzymatic hydrolysis performance [9].

As new types of catalytic materials, heteropolyacids (HPAs) and polyoxometalates (POMs) have attracted people’s attention due to their unique advantages of good acidity and redox properties [9,10,11]. In previous research, polyoxometalates were utilized as reagents or catalysts to control the extent of lignin oxidation during pulping or oxygen-based pulp bleaching processes [12,13,14]. However, some drawbacks which include low surface areas (1–5 m^2^⋅g^−1^), high solubility in polar solvents [15], and poor reusability [16] restrained their application. Considerable strategies were adopted to overcome these drawbacks. Among them, transition metals substitution was proven to effectively decrease their water solubility and enhance their reusability [17,18,19,20,21]. Immobilization of POMs on the supporters were also proven effective to enhance their performances, while different materials which include mesoporous silica, metal-organic frameworks (MOFs), metal oxides, and carbon materials were employed as supporters [22]. The modified POMs showed high reactivity and were successfully employed in the fields of electrochemistry, photochemistry, energy storage, and environmental protection [23].

In recent years, some researchers further explored the application of POMs in biorefinery processes. Xu et al. employed cesium substituted heterogeneous polyoxometalates as catalysts to improve birch lignin depolymerization in a mixture of CH_3_OH and H_2_O_2_ (4:1, *v*/*v*), and found that the catalyst was easily separated from products and possessed perfect reusability [17]. Ru/POM nanocatalyst also displayed high activity and selectivity in oxidative depolymerization and could be recovered easily [18]. Keggin-type polyoxometalate-supported Au nanoparticles were conducted as catalysts to converse cellobiose into gluconic acid in the presence of O_2_ when the highest cellobiose conversion and gluconic acid selectivity were ≥95% and 85%, respectively [19]. Zhang et al. proved that Mo-containing polyoxometalates were efficient for the conversion of cellulose into glycolic acid in water in O_2_ when the glycolic acid yield was about 49% under optimal conditions [20]. Wölfel et al. reported that vanadium-substituted polyoxometalate could oxidize glucose to formic acid under 343–363 K and an O_2_ pressure of 30 bar, while the yield of formic acid was about 50% [21].

Although DESs have been widely applied in the field of lignocellulosic biomass treatment, there are still many difficulties that need to be overcome. The processes usually need high temperatures and a long reaction time; the reusability of the catalysts still pose difficulties. The low extraction rate, low purity, and uneven molecular weight distribution of the obtained lignins significantly restrained the wide broad of this technology. To the best of our knowledge, there are still no reports that apply POMs as the catalyst to optimize the delignification process in DESs reaction systems [24,25]. In this research, Fe_3_O_4_-SiO_2_-V_3_ was prepared by deposited H_6_PMo_9_V_3_O_40_ on Fe_3_O_4_-SiO_2_ and employed as an easy recovery magnetic catalyst. FTIR, SEM, and EDX mapping were carried out to prove the success of the preparations. Lignins obtained from the CC-LA system and CC-LA-Fe-Si-V_3_ system were analyzed by Fourier-Transform Infrared (FT-IR), Gel Permeation Chromatography (GPC), and 2D-HSQC, respectively. Solid residues were also investigated by XRD. Reusability of the catalyst was also discussed.

## 2. Materials and Methods

### 2.1. Materials

We utilized corncob derived from Laiwu city, Shandong province. The raw corncob was washed with distilled water and ground into particles ranging from 40–60 mesh. Then, the powder was extracted by benzene-ethanol solution (benzene: ethanol = 2:1, volume ratio), dried at 60 °C for 6 h, and stored in a desiccator before application. The components of the corncob were analyzed according to the detection method of lignin and carbohydrate in biomass provided by NREL (National Renewable Energy Laboratory) [26]. Results indicated that it contains 40.12% cellulose, 32.74% xylan, 14.89% acid insoluble lignin, 1.96% acid soluble lignin, 3.67% ash, and 3.33% extraction.

### 2.2. Chemicals and Reagents

The chemicals and regents used were: Choline chloride, sodium phosphate dibasic dodecahydrate, diethyl ether, iron chloride hexahydrate, ferrous sulfate, sulfuric acid (98%), hydrochloric acid (36%), ammonium hydroxide solution, and tetraethyl orthosilicate (Sinopharm Chemical Reagent Co. Ltd., Shanghai, China); sodium metavanadate, sodium molybdate dihydrate, dimethyl sulfoxide, anhydride, pyridine, and oleic acid (Shanghai Macklin Biochemical Technology Co., Ltd., Shanghai, China); Tetrahydrofuran, ethanol, and toluene (Tianjin Hengxing Chemical Reagent Co., Ltd., Tianjin, China); lactic acid, and potassium bromide (Tianjin Kemiou Chemical Reagents Co. Ltd., Tianjin, China).

### 2.3. Preparation of DES

Deep eutectic solvent was synthesized according to the method described in previous literature [27]. Choline chloride and lactic acid were vacuum dried at 80 °C for 5 h before use. The molar ratio between choline chloride and lactic acid was set as 1:10 according to the results of our previous research. The mixture was stirred in an oil bath at 60 °C for 2 h until a homogeneous clear liquid was formed. 

### 2.4. Preparation of the Catalyst

The preparation of H_3+x_PMo_12−x_V_x_O_40_ (x = 1–3) was modified according to the method described in previous research [20,28]. Briefly, we put 50 mL NaVO_3_ (0.15 mol·L^−1^) and 50 mL Na_2_HPO_4_ (0.025 mol·L^−1^) into a 500-mL beaker in order. Then, 3 mL sulfuric acid (98%, *v*/*v*) and 100 mL Na_2_MoO_4_ solution (1.0 mol·L^−1^) were added into the beaker in sequence. Next, 42 mL sulfuric acid (98%, *v*/*v*) was added into the mixture slowly under vigorous stirring. The mixture was then extracted with ether to get red solids. We dissolved the red solids in 50 mL water, and put them in a vacuum dryer, while the solid product was placed in a dryer before use. Afterwards, 16.2 g FeCl_3_·6H_2_O and 8.34 g FeSO_4_·7H_2_O were dissolved in 125 mL distilled water, then we poured 185 mL boiling distilled water into the solution. Then, we added 33 mL ammonia into the mixture and stirred for 0.5 h. The precipitation was separated and washed with distilled water to obtain Fe_3_O_4_. Next, 8.0 g Fe_3_O_4_ was dispersed in 400 mL water, while oleic acid was added in at the same time to increase the uniformity of the solution. We then added 3.04 mL TEOS and 8.28 mL ammonia into the mixture. Finally, the particles were separated by magnetic force and washed with distilled water. Fe_3_O_4_-SiO_2_ was obtained by drying at 48 °C overnight. We added 0.6 g Fe_3_O_4_-SiO_2_ particles, 12.0 mL distilled water, 6.0 mL TEOS, 8.4 mL ethanol, and 1.2 mL of 0.01 M HNO_3_ into a 50-mL beaker; the mixture reacted under 40 °C until sol was formed. A certain amount of H_6_PMo_9_V_3_O_40_ was dissolved in 20.0 mL of ethanol and was slowly added into the sol. We kept stirring at 60 °C until a gel appeared; Fe_3_O_4_-SiO_2_-V_3_ can be obtained by drying the gel at 100 °C for 10 h [29,30].

### 2.5. The Extraction Process of Lignin 

Next, 2.0 g corncob powder was dispersed evenly into 60 g CC-LA (10:1). Then, a certain amount of the catalyst (0 and 0.06 g) was added into the reaction system and heated to the required temperature (90–130 °C) by oil bath. The mixture was kept under a certain temperature for 9–15 h.

**Figure 1:**
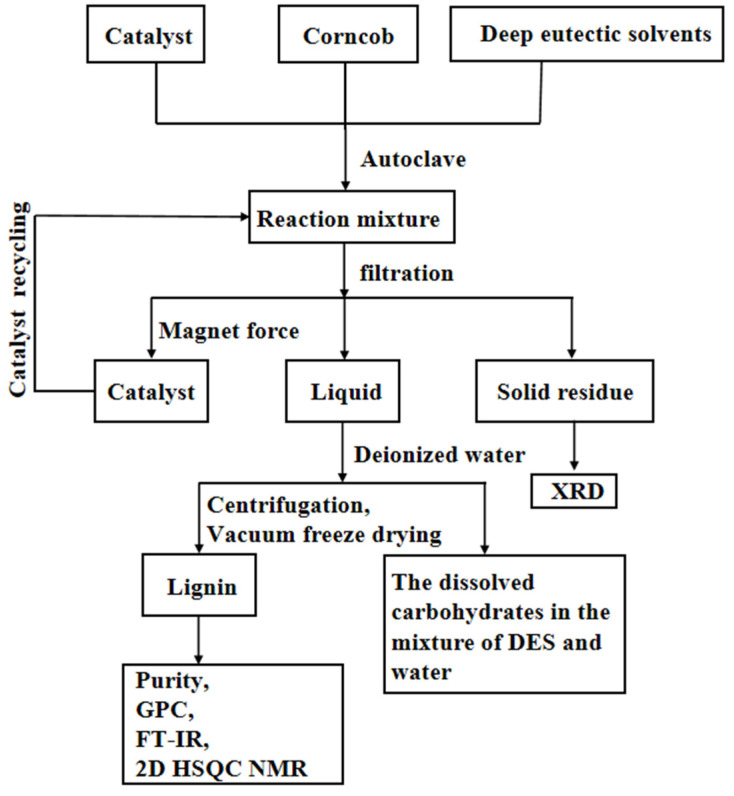
Flow diagram of thw lignin extraction process.

The mixture was filtered by G2. In addition, the solid residue was washed with anhydrous ethanol 3–5 times, then it was placed into an oven and dried under 90 °C until a constant mass was obtained (m_1_). The filtrate was treated by rotary evaporation to remove residual ethanol. Then, 2000 mL deionized water was added and fully stirred for 24 h; the precipitate was separated by centrifugation and washed with ethanol solution more than three times. The lignin powder (m_2_) was obtained by vacuum freeze-drying after centrifugation. Figure 1 exhibits the whole process.

(1) $$X=\frac{m_{1}}{m_{0}}\times 100\%$$

(2) $$Y=\frac{m_{2}}{m_{0}\times w}\times 100\%$$

X: percentage of solid residue quality, %;
Y: lignin extraction rate, %;
m_0_: quality of corncob, g;
m_1_: quality of solid residue, g.
m_2_: quality of lignin, g;
w: lignin content in corncob, %.

### 2.6. Samples Characterization and Calculation 

#### 2.6.1. Characterization of the Catalyst 

The surface microstructure of Fe_3_O_4_-SiO_2_ and Fe_3_O_4_-SiO_2_-V_3_ was observed by cold field emission scanning electron microscopy (SEM) Hitachi S4800. First, the double-sided conductive adhesive was glued to the copper base, so that the sample would be uniformly glued to the conductive adhesive. The sample was placed in the spraying room for gold spraying treatment, and then the sample was placed in the electron microscope observation room, and the magnification of scanning electron microscope was adjusted to observe the surface morphology of the sample.

EDX allows the determination of the content and distribution of each element on the surface of a sample, depending on the wavelength of the characteristic X-rays of the different elements. The EDX was used to determine the content of each element in Fe_3_O_4_-SiO_2_-V_3_ samples.

The diffraction patterns of untreated corncob and the solid residues after reaction were analyzed and determined by X-ray diffractometer (D8-Advance, Bruker, Germany). The scanning angle was set as 10–40°, 5°·min^−^^1^, the step size was 0.01°, the voltage was 40 kV, and the current was 40 mA.

#### 2.6.2. Characterization and Calculation of the Corncob and the Products

IR spectra were recorded on a PerkinElmer “Spectrum BX” spectrometer in the 4000–400 cm^−1^ region.

Lignin purity was carried out under following process: at first, 0.3 g of lignin and 3 mL of 72% concentrated sulfuric acid were added to a pressure resistant flask and reacted at 30 °C for 1 h. Then, 84 mL of deionized water was added to the flask and the reaction was carried out at 121 °C for 6 min. Next, lignin was filtered through an inorganic 0.22 μm filter membrane and detected by ion chromatography. The ratio of the sum of acid soluble lignin and acid insoluble lignin to the mass of lignin is the purity of the lignin.

The molecular weight of lignin was determined by gel permeation chromatograph (Waters e2695 Alliance HPLC, MA, USA). A total of 10 mg acetylated lignin was completely dissolved in 10 mL tetrahydrofuran solvent. The sample size was 10 μL, and the flow rate was 40 °C and 0.6 mL·min^−^^1^, respectively. The chromatographic column was Plgel Mixed-E (7.5 mm × 300 mm, 3 μm). The standard curve was determined by using the polystyrene standard with a different relative molecular weight.

The 2D-HSQC NMR spectra of the achieved lignin were conducted on AVANCE II 400 using 0.75 mL of DMSO d_6_ mixed with 0.1 mL pyridine d_5_ at room temperature.

The crystallinity index was calculated by the peak height method [31].

(3) $$\mathrm{CrI}=\frac{I_{002}-I_{\mathrm{am}}}{I_{002}}\times 100\%$$

I_002_ is the intensity of 002 lattice diffraction (2θ = 22.7°);
I_am_ is the intensity of amorphous section (2θ = 16.5°).

## 3. Results and Discussion

### 3.1. Characteristics of the Catalyst 

**Figure 2:**
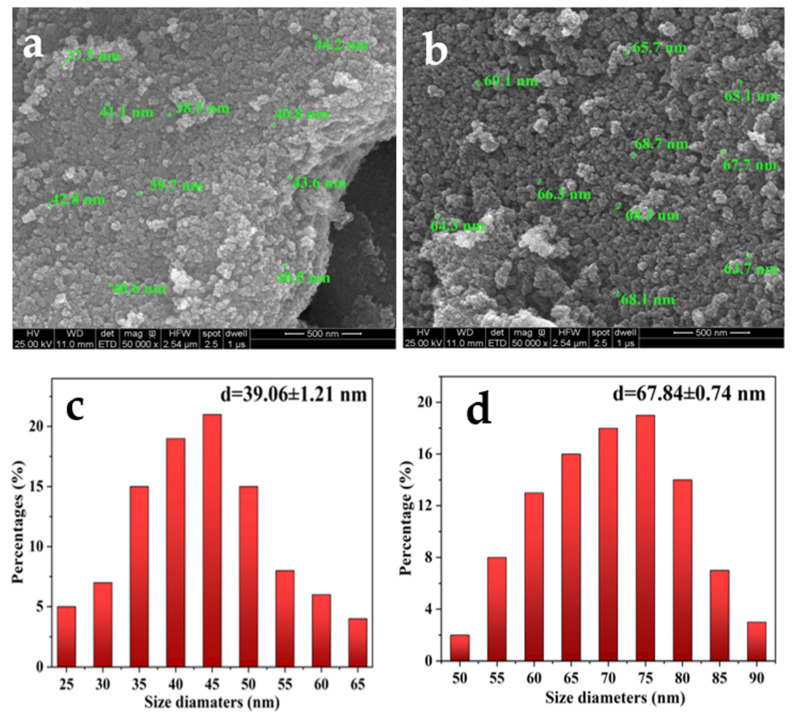

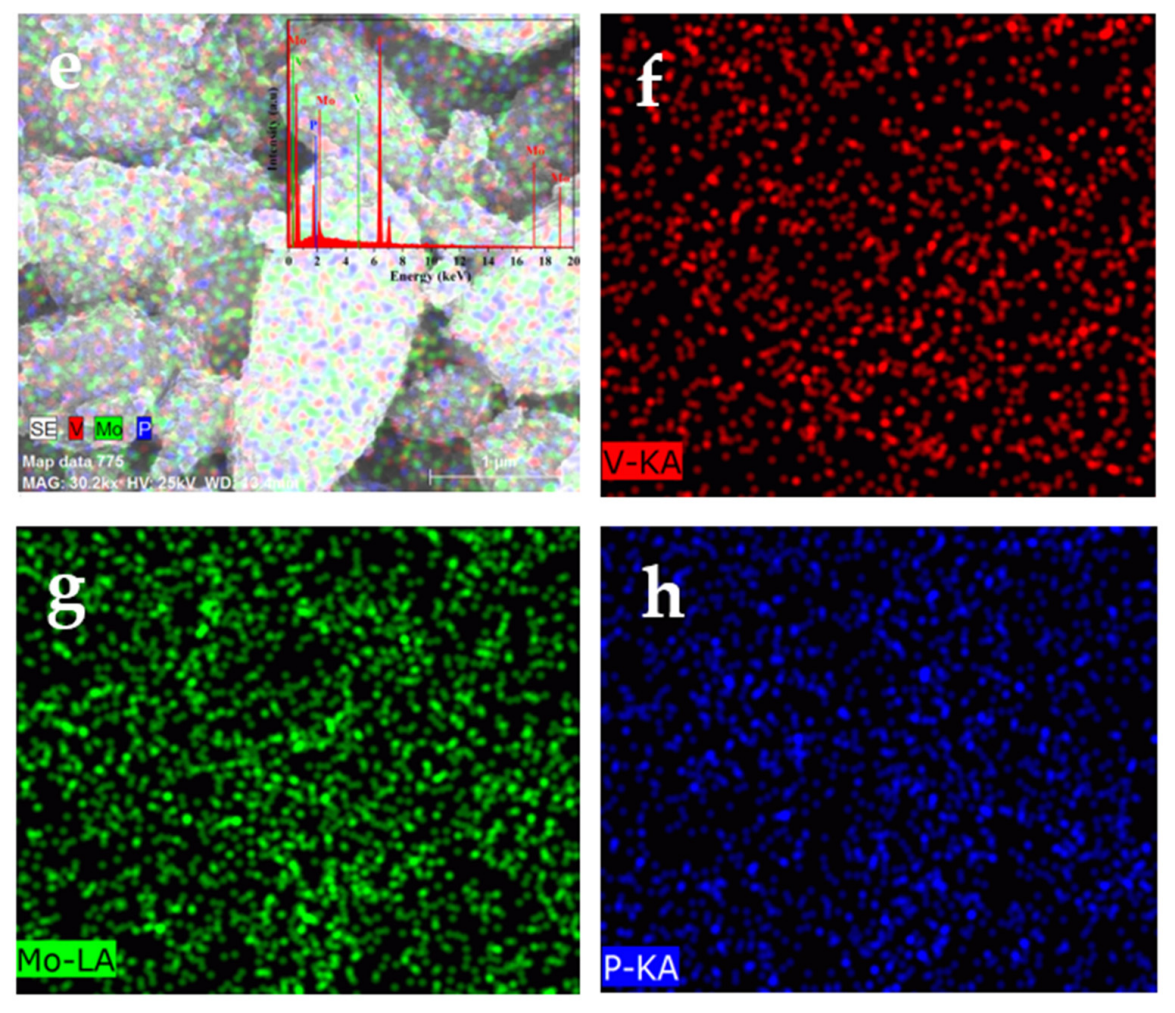
(**a**) SEM images of Fe_3_O_4_-SiO_2_; (**b**) SEM images of Fe_3_O_4_-SiO_2_-V_3_; (**c**) size distribution of Fe_3_O_4_-SiO_2_; (**d**) size distribution of Fe_3_O_4_-SiO_2_-V_3_; (**e**) EDX mapping of Fe_3_O_4_-SiO_2_-V_3_; (**f**) elemental mapping of V; (**g**) elemental mapping of Mo; (**h**) elemental mapping of P.

The SEM images of Fe_3_O_4_-SiO_2_ and Fe_3_O_4_-SiO_2_-V_3_ are shown in Figure 2a–d. The particle sizes of the products were measured by ImageJ 1.8.0. Results indicate the statistical particle size of Fe_3_O_4_-SiO_2_ is in the range of 35~50 nm. The statistical particle size of Fe_3_O_4_-SiO_2_-V_3_ is in the range of 60~80 nm. The precipitation of V_3_ on the surface of Fe_3_O_4_-SiO_2_ led to the increase of the particle size.

Figure 2e showed the EDX of Mo, V, and P distribution in Fe_3_O_4_-SiO_2_-V_3_. The appearance of P, Mo, and V in the EDX mapping of Fe_3_O_4_-SiO_2_-V_3_ proved that V_3_ loaded onto Fe_3_O_4_-SiO_2_ successfully. Furthermore, the results of Figure 2f–h also showed that V, Mo, and P were uniformly dispersed on the supporter.

### 3.2. Calculation of Crystallinity of Solid Residue

**Figure 3:**
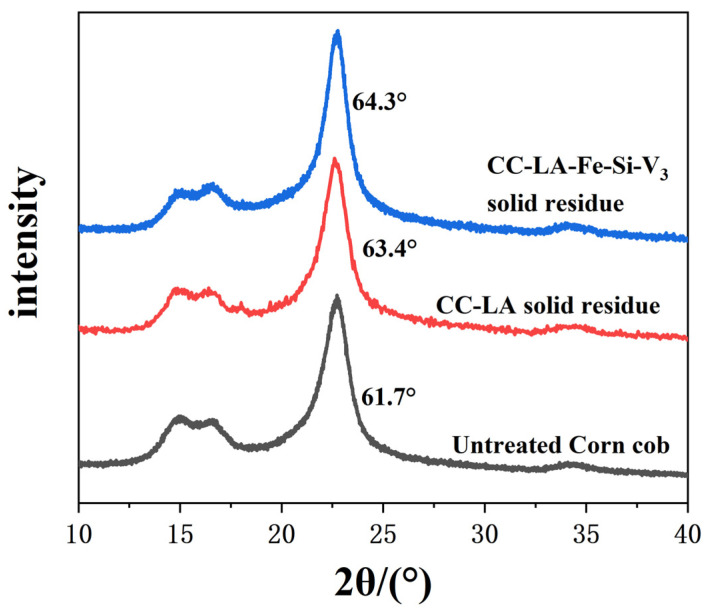
X-ray diffraction analysis spectra of various cellulose samples.

XRD spectra of untreated corncob and the solid residues after reaction were shown in Figure 3. The peak appeared 2θ = 16.5°and 22.7° indicated the cellulose structure of corncob should belong to type I. 

The crystallinity of untreated corncob was 61.7%, the crystallinity of CC-LA solid residue and CC-LA-Fe-Si-V_3_ solid residue was 63.4° and 64.3°, respectively. The crystallinity of the two solid residues treated by deep eutectic solvent and magnetic the catalyst was higher than that of the untreated corncob. However, the crystallinity of CC-LA-Fe-Si-V_3_ solid residue was higher than that of CC-LA solid residue. This might be attributed to the magnetic catalyst that destroyed the link between lignin and hemicellulose and removed lignin. The relative content of cellulose content of cellulose components led to the increase in the crystallinity of CC-LA-Fe-Si-V_3_ solid residue.

### 3.3. Characterization of Lignin

Table 1 shows the purity changes of CC-LA lignin and CC-LA-Fe-Si-V_3_ lignin at different temperatures. When the reaction temperature increased from 90 °C to 120 °C, there were little fluctuate occurred for both reaction systems. The variation range of purity for CC-LA lignin was 85.6%–86.8%. Meanwhile, it was 97.1–98.3% for CC-LA-Fe-Si-V_3_ lignin. When the temperature increased to 130 °C, the lignin purities for both reaction systems decreased. The purity of CC-LA-Fe-Si-V_3_ lignin was significantly higher than that of CC-LA lignin. This proved that the application of magnetic the catalyst was beneficial for improving the purity of lignin.

Table 2 displayed the variation of the heavy average molecular weight (M_w_) and number average molecular weight (M_n_) of CC-LA lignin and CC-LA-Fe-Si-V_3_ lignin with the reaction temperature. Results showed that the M_w_ and M_n_ of both reaction systems had a declining tendency with the increase of the reaction temperature. The M_w_ of CC-LA lignin decreased from 1752 g·mol^−1^ to 1366 g·mol^−1^ and the M_n_ decreased from 900 g·mol^−1^ to 731 g·mol^−1^. The M_w_ of CC-LA-Fe-Si-V_3_ lignin decreased from 1482 g·mol^−1^ to 1142 g·mol^−1^ and the M_n_ decreased from 773 g·mol^−1^ to 654 g·mol^−1^. This could be attributed to the fact that the increase in reaction temperature facilitated the breaking of the β-O-4 bond [32]. The M_w_ and M_n_ of CC-LA-Fe-Si-V_3_ lignin were lower than those of CC-LA lignin, regardless of the change in reaction temperature. This might have been because of the CC-LA-Fe-Si-V_3_ reaction system facilitating the cleavage of the lignin-carbohydrate complex (LCC) [33].

The polydispersity index (PDI) of lignin was calculated by the weight average molecular weight and number average molecular weight ratio (M_w_/M_n_) of lignin. The polydispersity index of CC-LA lignin was in the range of 1.93~1.87. The polydispersity index of CC-LA-Fe-Si-V_3_ lignin ranged from 1.92 to 1.75. Compared with CC-LA lignin, CC-LA-Fe-Si-V_3_ lignin had a lower polydispersity index, indicating that the molecular distribution of CC-LA-Fe-Si-V_3_ lignin was relatively narrow.

**Figure 4:**
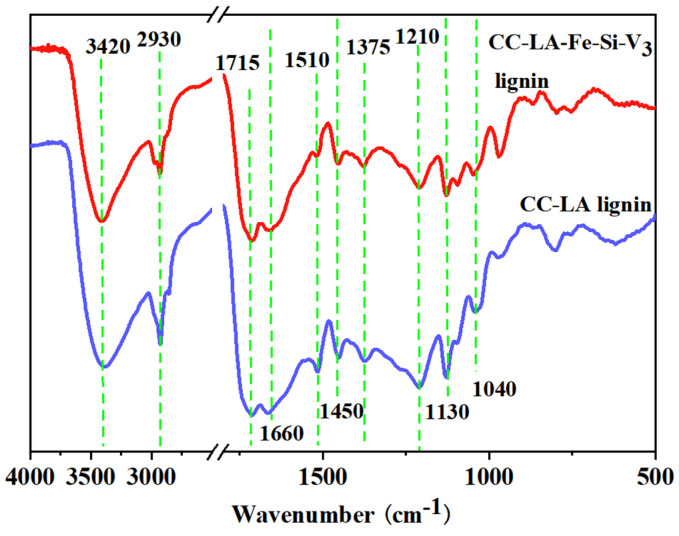
FT-IR analysis of CC-LA lignin and CC-LA-Fe-Si-V_3_ lignin.

Figure 4 shows the FT-IR spectra results of the lignins obtained from both reaction systems. CC-LA-Fe-Si-V_3_ lignin presented similar absorbance bands with those of CC-LA lignin. Peaks that appeared at 3420 cm^−1^ and 2930 cm^−1^ corresponded to the stretching mode of O-H and the aliphatic C-H stretching related to the methoxy group, respectively [34]. The bands presented at 1715 cm^−1^ and 1510 cm^−1^ were associated with C-O stretching in unconjugated carbonyl and carboxyl groups and skeletal and stretching vibrations of benzene rings. The absorption at 1450 cm^−1^ was associated with aromatic ring vibration. The absorption at 1375 cm^−1^ was associated with C-C stretching of the S unit.

In addition, the characteristic signal of the skeletal and stretching vibrations of benzene rings and lignin structures occurred at 1510 cm^−1^ [35]. Moreover, the signal of C=O stretching vibration of the guaiacyl (G) unit occurred at 1210 cm^−1^ [36]. It was noticed that the signal was clearly more distinct in CC-LA lignin than in CC-LA-Fe-Si-V_3_ lignin, which indicated that the addition of Fe_3_O_4_-SiO_2_-V_3_ led more aromatic rings to be decomposed and to the destruction of more guaiacyl (G) units. In addition, the peaks exhibited at 1130 cm^−1^ and 1040 cm^−1^ should belong to the characteristic signals of C-H stretching vibration of syringyl units and C-H bending vibration of guaiacyl units in a lignin structure [37].

**Figure 5:**
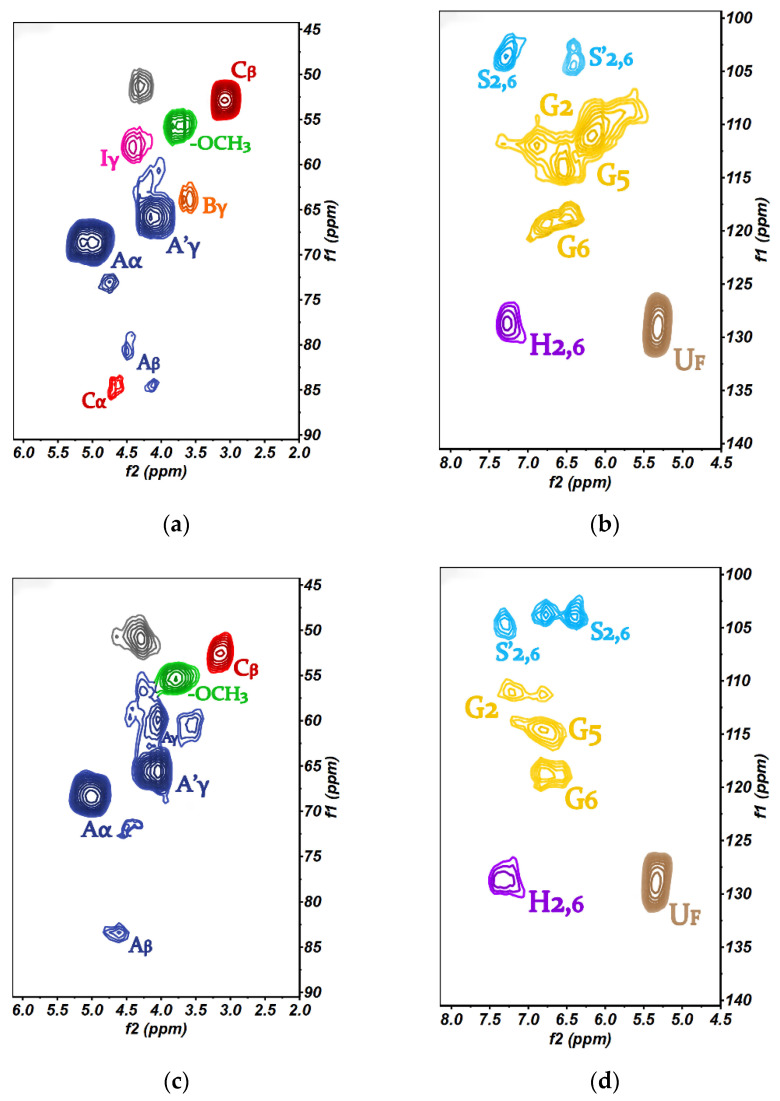

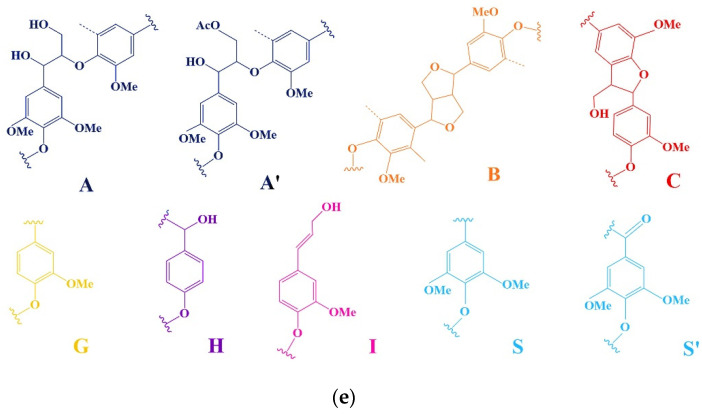
(**a**,**c**) The 2D-HSQC NMR spectra of CC-LA Lignin; (**b**,**d**) The 2D-HSQC NMR spectra of CC-LA-Fe-Si-V_3_ lignin; (**e**) The structure of various functional groups in 2D-HSQC NMR spectra.

To elucidate the characteristics of the CC-LA-Fe-Si-V_3_ lignin more clearly, the structure and chemical composition of lignin as extracted by 2D-HSQC NMR were analyzed. The 2D-HSQC spectra of CC-LA lignin and CC-LA-Fe-Si-V_3_ lignin are shown in Figure 5. In the 2D-HSQC NMR spectra of the (δC/δH, 45~90 ppm/2.0~6.0 ppm) region, there mainly is a methoxy group with a β-O-4 structure (A) and β-O-4 acetylated structure (A′) in CC-LA lignin. The α position of β-O-4 linkages appeared at (δC/δH,68.41/5.02 ppm) and (δC/δH,72.96/4.72 ppm). The β position of β-O-4′ linkages was exhibited at (δC/δH, 80.79/4.56 ppm) and (δC/δH, 84.21/4.25 ppm) [38,39]. The correlation of C_γ_-H_γ_ in the γ-acylated lignin unit (A′_γ_) was observed at (δC/δH, 65.67/4.18 ppm). In addition, the spectra of CC-LA lignin components also showed a p-hydroxycinnamyl alcohol end group, phenylcoumaran substructures (Bγ), and β-β′ resinol substructures (C_β_).

In the 2D-HSQC NMR spectra of CC-LA-Fe-Si-V_3_ lignin, the α position of β-O-4 linkages was exhibited at (δC/δH, 68.31/5.02 ppm) and (δC/δH, 72.21/4.52 ppm). The β position of β-O-4′ linkages appeared at (δC/δH, 83.56/4.61 ppm). The correlation of C_γ_-H_γ_ in a γ-acylated lignin unit (A′γ) was observed at (δC/δH, 65.64/4.05 ppm). In addition, resinol substructures appeared at (δC/δH, 52.93/3.14 ppm) and a p-hydroxycinnamyl alcohol end group (I) was observed at (δC/δH, 57.03/4.27 ppm). CC-LA lignin contains the C_γ_-H_γ_ phenylcoumaran substructures (δC/δH 63.71/3.61). CC-LA-Fe-Si-V_3_ lignin has the C_γ_-H_γ_ in γ-hydroxylated β-O-4′ substructures (δC/δH, 60.66/3.58 ppm) [40]. This is the main difference between the CC-LA lignin and CC-LA-Fe-Si-V3 lignin.

In the 2D-HSQC NMR spectra of the (δC/δH, 100~140 ppm/4.5~8.0 ppm) region, there are S and G units of lignin. In the 2D-HSQC NMR spectra of CC-LA lignin, a C_2,6_-H_2,6_ signal of S units was found at (δC/δH, 104.63/6.41 ppm) and (δC/δH, 104.75/7.45 ppm). In the 2D-HSQC NMR spectra of CC-LA-Fe-Si-V_3_ lignin, a C2,6-H2,6 signal of S units was observed at (δC/δH,104.19/6.59 ppm) and (δC/δH, 105.31/7.21 ppm) [41,42]. In the 2D-HSQC NMR spectra of CC-LA lignin, C_2_-H_2_, C_5_-H_5_, and C_6_-H_6_ of G unit were observed at (δC/δH, 111.78/7.03 ppm), (δC/δH, 115.47/6.68 ppm), and (δC/δH, 119.61/6.73 ppm), respectively. In the 2D-HSQC NMR spectra of CC-LA-Fe-Si-V_3_ lignin, C_2_-H_2_, C_5_-H_5_, and C_6_-H_6_ of G unit were observed at (δC/δH, 111.89/7.34 ppm), (δC/δH, 115.21/6.43 ppm), and (δC/δH, 119.32/6.65 ppm), respectively [43]. In addition, strong linoleic acid signals were found in the spectrum.

CC-LA lignin and CC-LA-Fe-Si-V_3_ lignin samples possess an intact structure. More C_2_-H_2_ and C_5_-H_5_ in G units of CC-LA-Fe-Si-V_3_ lignin were destroyed than CC-LA lignin. This further proves the results of FT-IR spectra. Fe_3_O_4_-SiO_2_-V_3_ has both destructive and protective functions on the different structures of lignin. In the 2D-HSQC NMR spectra of CC-LA-Fe-Si-V_3_ lignin, C_γ_-H_γ_ in phenylcoumaran substructures were protected. Additionally, C_γ_-H_γ_ in γ-hydroxylated β-O-4′ substructures was destroyed.

### 3.4. Possible Mechanism of the Catalysis Process

**Figure 6:**
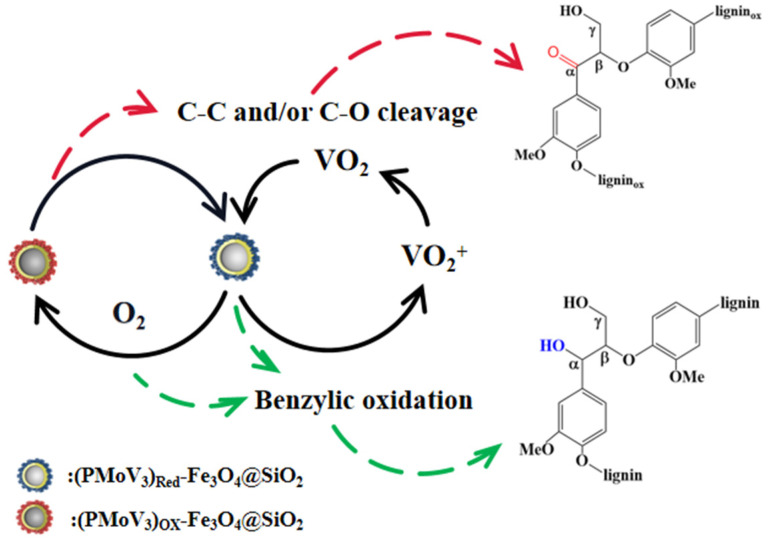
Possible mechanism of the delignification process.

The mechanism of the delignification process catalyzed by POMs mainly affects their swift reversible multi-electron redox transformation property and variable acid-base and redox properties, which result in high conversion and selectivity through benzylic alcohol oxidation [11,44]. Figure 6 indicates the might mechanism of the delignification process of the CC-LA-PMoV_3_ reaction system. 

In acidic solutions, PMoV_3_ could disproportionate and provide species with a number of vanadium atoms which are different from the original molecular structure [45]:

(4) $${\mathrm{PMoV}}_{3}↔{\mathrm{PMoV}}_{2}+VO_{2}^{+}$$

(5) $${\mathrm{PMoV}}_{2}↔{\mathrm{PMoV}}_{1}+VO_{2}^{+}.$$

The anodic potentials of lignin structural units which bear phenolic hydroxyl groups are usually in the range +0.40 to +0.60 V [46]. Meanwhile, the reduction potentials of PMoV_n_ (*n* = 1–3) are in the range +0.68 to +0.778 V, and the oxidation potential of VO_2_^+^ is about 0.87 V. Thus, the redox potential of the reaction system is enough to satisfy the thermodynamic conditions for the catalytic oxidation of the lignin in corncob. During the delignification process, the C_α_ structure which possesses a hydroxy unit can be activated by the catalysts and selectively oxidized to ligninox, then it could be further oxidized by VO_2_^+^ and promote the cleavage of the benzylic units [21]. Secondly, the reduced PMoV_3_ and VO_2_ could be turned to oxidized PMoV_3_ and VO_2_^+^ with the assistance of O_2_. During this process, the cleavage of β-O-4 ether and C_α_-C_β_ linkages would occur under the assistance of acidic conditions. A related mechanism could be described as Equations (6)–(10).

(6) $$\mathrm{lignin}+{\mathrm{PMoV}}_{3}+H^{+}\to {\left({\mathrm{PMoV}}_{3}\right)}_{\mathrm{red}}+{\mathrm{lignin}}_{ox}\;$$

(7) $$\mathrm{lignin}+VO_{2}^{+}\to {\mathrm{lignin}}_{ox}+V\left(IV\right)+H^{+}$$

(8) $${\mathrm{lignin}}_{\mathrm{ox}}+O_{2}\to {\mathrm{lignin}}_{\mathrm{ox}}^{*}\;\left(\mathrm{activated}\right)$$

(9) $${\mathrm{lignin}}_{ox}^{*}\;\left(\mathrm{activated}\right)+V\left(\mathrm{IV}\right)\to \mathrm{depolymerized}\;\mathrm{lignin}\;\mathrm{fractions}+V\left(V\right)$$

(10) $${\left({\mathrm{PMoV}}_{3}\right)}_{\mathrm{red}}+O_{2}\to {\mathrm{PMoV}}_{3}$$

### 3.5. Reusability of the Magnetic Catalyst

After the reaction was separated and recovered, the supported the catalyst and the mixed solution of ethanol and water (ethanol: water = 1:9) were used for centrifugal washing and drying. The lignin extraction rates from the first to the sixth reaction of Fe_3_O_4_-SiO_2_-V_3_ were 98.1%, 96.4%, 94.9%, 93.6%, 93.1%, and 92.8%, respectively. After six reactions, the supported catalyst still had high activity, and the extraction rate of lignin decreased by only 5.3%. By comparing the structure of Fe_3_O_4_-SiO_2_-V_3_ before and after the reaction, it was found that the magnetic catalyst Fe_3_O_4_-SiO_2_-V_3_ had no significant change after repeated use, showing high stability.

## 4. Conclusions

Both the CC-LA reaction system and the CC-LA-Fe-Si-V_3_ reaction system were used to selectively separate lignin from corncob. The CC-LA-Fe-Si-V_3_ reaction system has more advantages than the CC-LA reaction system. The extraction rate of lignin increased from 71.65% to 98.13%, while the purity of lignin increased from 85.62% to 97.09%. The CC-LA-Fe-Si-V_3_ reaction system has both destructive and protective functions affecting the different structures of lignin. The CC-LA-Fe-Si-V_3_ reaction system proposed in the present work could improve the extraction of lignin. CC-LA lignin and CC-LA-Fe-Si-V_3_ lignin samples possess an intact structure. The aromatic rings and guaiacol units in CC-LA-Fe-Si-V_3_ lignin were clearly destroyed. C_γ_-H_γ_ in phenylcoumaran substructures in CC-LA-Fe-Si-V_3_ lignin was protected, and C_γ_-H_γ_ in γ-hydroxylated β-O-4′ substructures was destroyed. Compared to CC-LA lignin, CC-LA-Fe-Si-V_3_ lignin has a higher purity, lower molecular weight, and more concentrated distribution. CC-LA-Fe-Si-V_3_ lignin showed more potential application values in chemical industries. 

## Figures and Tables

**Table 1:** Purities of CC-LA lignin and CC-LA-Fe-Si-V_3_ lignin.

	90 °C	100 °C	110 °C	120 °C	130 °C
CC-LA lignin (%)	86.82	86.5	86.1	85.62	82.35
CC-LA-Fe-Si-V_3_ lignin (%)	98.25	97.75	97.6	97.09	95.34

**Table 2:** Average molecular weights and polydispersity indexes (PDI) of different lignins.

		90 °C	100 °C	110 °C	120 °C	130 °C
CC-LA lignin	M_w_ (g·mol^−1^)	1752	1521	1461	1384	1366
	M_n_ (g·mol^−1^)	909	795	776	740	731
	PDI	1.93	1.91	1.88	1.87	1.87
CC-LA-Fe-Si-V_3_ lignin	M_w_ (g·mol^−1^)	1482	1451	1371	1336	1142
	M_n_ (g·mol^−1^)	773	772	751	738	654
	PDI	1.92	1.88	1.83	1.81	1.75

## Data Availability

Not applicable.
